# Predictive Value of Cumulative Blood Pressure for All-Cause Mortality and Cardiovascular Events

**DOI:** 10.1038/srep41969

**Published:** 2017-02-07

**Authors:** Yan Xiu Wang, Lu Song, Ai Jun Xing, Ming Gao, Hai Yan Zhao, Chun Hui Li, Hua Ling Zhao, Shuo Hua Chen, Cheng Zhi Lu, Shou Ling Wu

**Affiliations:** 1 Department of Cardiology, Tianjin First Center Hospital, Clinical Medical College of Tianjin Medical University, Tianjin, China; 2 Department of Cardiology, Kailuan Hospital, North China University of Science and Technology, Tangshan, China; 3 Graduate school, North China University of Science and Technology, Tangshan, China; 4 Department of Health Care Center, Kailuan Hospital, North China University of Science and Technology, Tangshan, China

## Abstract

The predictive value of cumulative blood pressure (BP) on all-cause mortality and cardiovascular and cerebrovascular events (CCE) has hardly been studied. In this prospective cohort study including 52,385 participants from the Kailuan Group who attended three medical examinations and without CCE, the impact of cumulative systolic BP (cumSBP) and cumulative diastolic BP (cumDBP) on all-cause mortality and CCEs was investigated. For the study population, the mean (standard deviation) age was 48.82 (11.77) years of which 40,141 (76.6%) were male. The follow-up for all-cause mortality and CCEs was 3.96 (0.48) and 2.98 (0.41) years, respectively. Multivariate Cox proportional hazards regression analysis showed that for every 10 mm Hg·year increase in cumSBP and 5 mm Hg·year increase in cumDBP, the hazard ratio for all-cause mortality were 1.013 (1.006, 1.021) and 1.012 (1.006, 1.018); for CCEs, 1.018 (1.010, 1.027) and 1.017 (1.010, 1.024); for stroke, 1.021 (1.011, 1.031) and 1.018 (1.010, 1.026); and for MI, 1.013 (0.996, 1.030) and 1.015 (1.000, 1.029). Using natural spline function analysis, cumSBP and cumDBP showed a J-curve relationship with CCEs; and a U-curve relationship with stroke (ischemic stroke and hemorrhagic stroke). Therefore, increases in cumSBP and cumDBP were predictive for all-cause mortality, CCEs, and stroke.

Hypertension is the most common chronic disease, and the most important risk factor for cardiovascular disease^1^. Stroke and myocardial infarction (MI) are the main complications of hypertension that can lead to death^1^,^2^,^3^,^4^. The Framingham Study showed that starting from 115/75 mm Hg, the risk for cardiovascular events increases following the increase in blood pressure (BP)^5^. It takes time for exposure to high BP to become a risk of all-cause mortality and cardiovascular and cerebrovascular events (CCEs); and there are many factors affecting BP, such as age, diet, lifestyle, and use of antihypertensive drugs. Therefore, using a single BP measurement to predict all-cause mortality and the occurrence of CCE is not reliable.

Cumulative exposure is calculated as the product of the dose level and the exposure time and has been used to predict the impact of exposures on the target organ. Since Doll and Hill first proposed that high cumulative exposure to smoking is associated with lung cancer^6^,^7^, it has been suggested that cumulative exposure to high blood sugar level increases the risk of complications of diabetes^8^, cumulative exposure to high cholesterol level increases the risk for coronary heart disease^9^, and cumulative exposure to high BP is associated with kidney damage^10^. However, there is hardly any study on the predictive value of cumulative exposure to elevated BP on all-cause mortality and the occurrence of CCEs. In this study, we used data collected from the Kailuan Study (Trial identification: ChiCTR–TNC–11001489; Trial registration site: http://www.chictr.org.cn/index.aspx; Registration number: 11001489) and analyzed the predictive value of cumulative BP for all-cause mortality and CCEs.

## Methods

### Study Population

From 2006 to 2007, a general medical examination was carried out for the serving and retired employees of the Kailuan Group by 11 hospitals in Kailuan (Hebei, China). Subsequent medical examinations took place in 2008–2009 (the second), 2010–2011 (the third), and 2012–2013 (the fourth). The same groups of medical professionals from the first examination performed the following examinations on the same groups of participants using the same medical facilities. The medical examinations and the anthropometric and laboratory measurements were the same. For all participants, the time intervals between each examination were similar. For the current study, data from the first three examinations were analyzed. The current study was approved by the Ethics Committee of the Kailuan General Hospital, and it was in accordance with the Declaration of Helsinki.

### The Inclusion and Exclusion Criteria

Participants were eligible for the current study if they were in the first, the second, and the third examination; aged ≥18 years; had records of BP measurements for all three examinations; agreed to participate; and provided written informed consent. Participants were excluded if they had a history of MI, a history of stroke, missing records of BP measurements, and did not agree to participate in this study.

### Data Collection

Details of the collection of epidemiological data and anthropometric and laboratory measurements were published previously^11^. BP was measured between 7–9 am in the morning of the medical examination. No coffee, tea, or smoking was allowed within 30 min of BP measurement. The participants were asked to sit quietly with their back supported for 15 min prior to BP measurement. Calibrated mercury sphygmometers were used to measure BP in the right brachial artery. The first and the fifth Korotkoff sound were used for systolic BP (SBP) and diastolic BP (DBP), respectively. BP readings were taken for 3 consecutive times with 1–2 min interval between the measurements, and the average of the three readings was used. Smoking was defined as having smoked at least one cigarette every day for the previous year. Drinking was defined as having 100 mL strong spirit (alcohol content > 50%) daily for at least the previous year. Exercise was defined as having ≥3 exercise sessions weekly with each session lasting at least 30 min.

The follow-up period started from the day after the participants had their third medical examination in 2010–2011. All-cause mortality was defined as deaths due to any causes except accidents during follow-up. CCEs were defined as MI and stroke. The last day of follow-up for CCEs was 31 December 2013, and for all-cause mortality, the last day of follow-up was 31 December 2014. Information on deaths and CCEs was obtained annually through the Social Security Information System of Kailuan.

### Definitions

Cumulative blood pressure (cumBP) was calculated as described by Zemaitis *et al*.^10^. cumBP = [(BP_1_ + BP_2_)/2 × time_1–2_] + [(BP_2_ + BP_3_)/2 × time_2–3_], where BP_1_, BP_2_, and BP_3_ were measurements of BP recorded from the first, the second, and the third medical examination; time_1-2_ and time_2-3_ were the time intervals between the first and the second, and the second and the third BP measurements. cumBP included cumulative SBP (cumSBP) and cumulative DBP (cumDBP), which were calculated similarly. Standardized cumBP (ScumBP) was calculated as cumBP/(time_1-2_ + time_2-3_), including standardized cumulative SBP (ScumSBP) and standardized cumulative DBP (ScumDBP).

### Statistical Analysis

Data input was carried out by trained personnel of each participating hospital. The database (Oracle Database 10.2) was hosted at the Kailuan General Hospital. SPSS 13.0 was used for data analysis. For continuous parameters following a normal distribution, mean ± standard deviation (SD) was used; one-way analysis of variance (ANOVA) and pairwise comparison was used for comparison between groups. N (%) was used for discrete data and chi-square test was used for comparison between groups. Life table was used to calculate the cumulative incidence of endpoint events (all-cause mortality and CCEs) by cumSBP; and the differences in cumulative incidence were tested by log-rank test. Multivariate Cox proportional hazards regression model and the natural spline function were used to further analyze the risk (hazard ratios [HRs] and 95% confidence intervals [CIs]) for all-cause mortality and CCEs by cumSBP and cumDBP. Model1 was adjusted for age and sex; model 2 was further adjusted for baseline SBP/DBP, body mass index (BMI), fasting glucose(FBG), high density lipoprotein cholesterol(HDL-C),exercise, smoking, drinking, and antihypertensive drugs use; model 3 was further adjusted for salt intake, estimated glomerular filtration rate (eGFR), lipid-lowering drugs use, diabetes medications, and number of antihypertensive medications. We also conducted several sensitivity analyses to test the robustness of our findings. We repeated our aforementioned analysis by excluding individuals with hypertension, those who died within 1 year after the third annual medical examination, those who without atrial fibrillation, or those paticipants of untreated hypertensive, to examine whether the relation between cumSBP/cumDBP and all endpoints were altered. *P* < 0.05 was considered statistically significant (two-tailed).

Due to different follow-up time of the participants, the calculated cumBP might have an impact on the findings. Therefore, a sensitivity analysis was carried out for the COX regression, in which cumBP was standardized and the HRs were calculated again using the ScumBP.

## Results

A total of 57,927 participants had taken all three medical examinations in 2006–2007, 2008–2009, and 2010–2011. During this period, 3791 cases of MI and stroke were reported. 1751 participants had missing records of BP measurements. The current study included data from 52,385 participants.

### Patient Characteristics

The mean ± SD age was 48.82 ± 11.77 years for the study population, 40,141 (76.6%) of the participants were male. The four groups of cumSBP (mm Hg·year) were: cumSBP < 120 mm Hg × 6 year for the first group; 720 ≤ cumSBP < 130 mm Hg × 6 year for the second group; 780 ≤ cumSBP < 140 mm Hg × 6 year for the third group; and cumSBP ≥840 for the fourth group. From the first to the fourth group, significant increases were found for age, heart rate, baseline SBP, SBP (Visit 2,Visit 3), baseline DBP, DBP (Visit 2,Visit 3), body mass index (BMI), fasting blood glucose (FBG), total cholesterol (TC), high density lipoprotein cholesterol (HDL-C), low-density lipoprotein cholesterol (LDL-C), salt intake, estimated glomerular filtration rate (eGFR), drinking, exercise, hypertension, antihypertensive drugs, use lipid-lowering drugs use, and diabetes medications (P < 0.05, respectively) (Table 1).

### cumSBP and Endpoint Events

**Figure 1:**
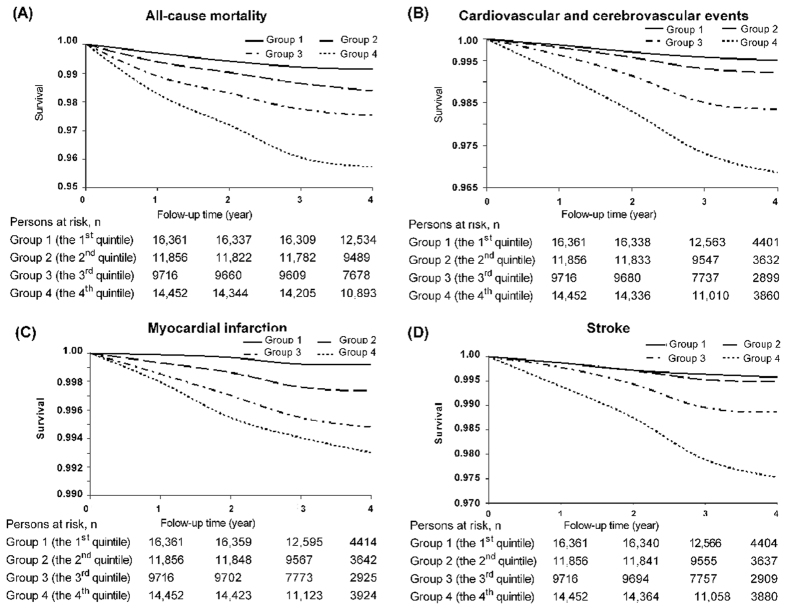
The Survival Curve of the Study Population. (**A**) All-cause mortality, (**B**) Cardiovascular and cerebrovascular events, (**C**) Myocardial infarction, and (**D**) Stroke.

The mean ± SD follow-up time for all-cause mortality was 3.96 ± 0.48 years; for CCEs, it was 2.98 ± 0.41 years. A total of 1048 deaths (all-cause) and 660 CCEs occurred, including 166 cases of MI and 497 cases of stroke (3 cases also with MI). In the study population, the incidence of all-cause mortality, CCEs, and stroke increased with the increase in cumSBP, and the differences among the cumSBP groups were significant (log-rank test, *P* < 0.05, respectively) (Table 2 and Fig. 1).

Using all-cause mortality, CCEs, stroke, or MI as the dependent variable, cumSBP or cumDBP as the independent variable, the risk for endpoint events was calculated using Cox proportional hazards model and the natural spline function. After adjusted for other confounders including sex, age, baseline SBP, baseline DBP, BMI, FBG, HDL-C, exercise, smoking, drinking, antihypertensive drugs use, salt intake, eGFR, lipid-lowering drugs use, diabetes medications, and number of antihypertensive medications, for every 10 mm Hg·year increase in cumSBP, or every 5 mm Hg·year increase in cumDBP. the HRs were 1.013 (95% confidence interval: 1.006, 1.021) and 1.012 (1.006, 1.018) for all-cause mortality, 1.018 (1.010, 1.027) and 1.017 (1.010, 1.024) for CCEs, 1.013 (0.996, 1.030) and 1.015 (1.000, 1.029) for MI, 1.021 (1.011, 1.031) and 1.018 (1.010, 1.026) for stroke (Tables 3 and 4). The relevant HRs of cumSBP were 1.021 (95% CI: 1.008, 1.033), 1.030 (95% CI: 1.013, 1.046), 1.040 (95% CI: 1.010, 1.071), and 1.029 (95% CI: 1.009, 1.048), cumDBP were 1.017 (95% CI: 1.006, 1.027), 1.027 (95% CI: 1.014, 1.040), 1.038 (95% CI: 1.015, 1.061) and 1.025(95% CI: 1.009, 1.040), among nonhypertension participants, respectively (Supplementary Tables 3 and 4). The relevant cumSBP were 1.015 (1.007, 1.023), 1.018 (1.010, 1.027), 1.013 (0.995, 1.030), and 1.021 (1.011, 1.030), cumDBP were 1.014 (1.007, 1.021), 1.017 (1.010, 1.024), 1.015 (1.000, 1.029), and 1.018 (1.010, 1.026) among participants of excluded those who died within 1 year after the third annual medical examination, respectively (Supplementary Tables 5 and 6). The relevant cumSBP were 1.013 (1.005, 1.020), 1.018 (1.010, 1.027), 1.012 (0.995, 1.030), and 1.021 (1.011, 1.031), cumDBP were 1.011 (1.005, 1.018), 1.017 (1.010, 1.023), 1.015 (1.001, 1.028), and 1.018 (1.010, 1.026) among participants of without atrial fibrillation, respectively (Supplementary Table 7). The relevant cumSBP were 1.008 (0.997, 1.019), 1.013 (1.001, 1.025), 1.002 (0.977, 1.027), and 1.017(1.003, 1.030), cumDBP were 1.008 (0.998, 1.017), 1.012 (1.003, 1.022), 1.003 (0.983, 1.024), and 1.015 (1.004, 1.025) among untreated hypertensive (Supplementary Table 8).

In multivariable Cox models, we adjusted for aforementioned covariates. In adjusted models, compared with baseline SBP, the predictive value of cumSBP for both all-cause mortality and stroke was better (*P* < 0.01), but not for CCEs and MI (*P* > 0.05). Compared with baseline DBP, the predictive value of cumDBP for both CCEs and stroke was better (*P* < 0.01), but not for all-cause mortality and MI (*P* > 0.05) (Table 5).

**Figure 2:**
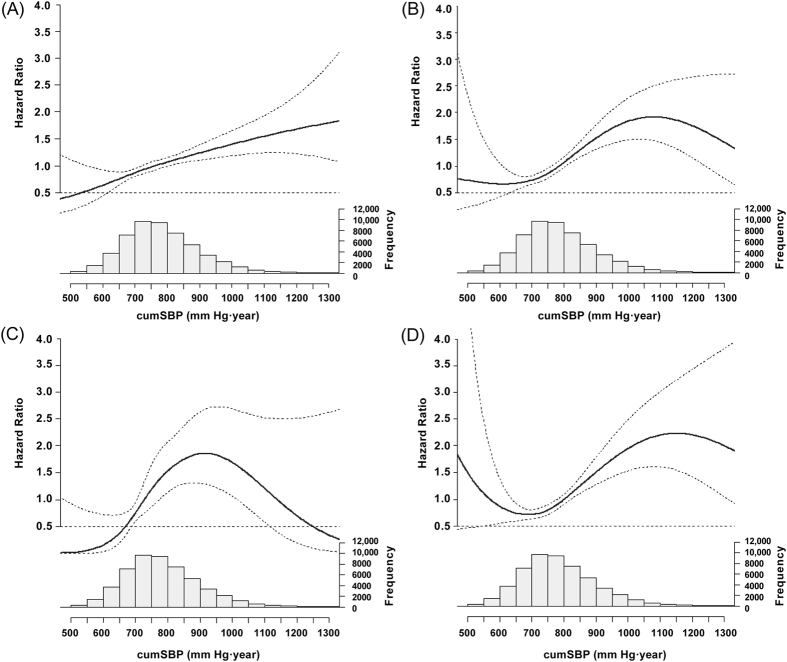
The Relationship Between cumSBP and Endpoint Events in the Study Population. (**A**) All-cause mortality, (**B**) Cardiovascular and cerebrovascular events, (**C**) Myocardial infarction, and (**D**) Stroke cumSBP, cumulative systolic blood pressure.

**Figure 3:**
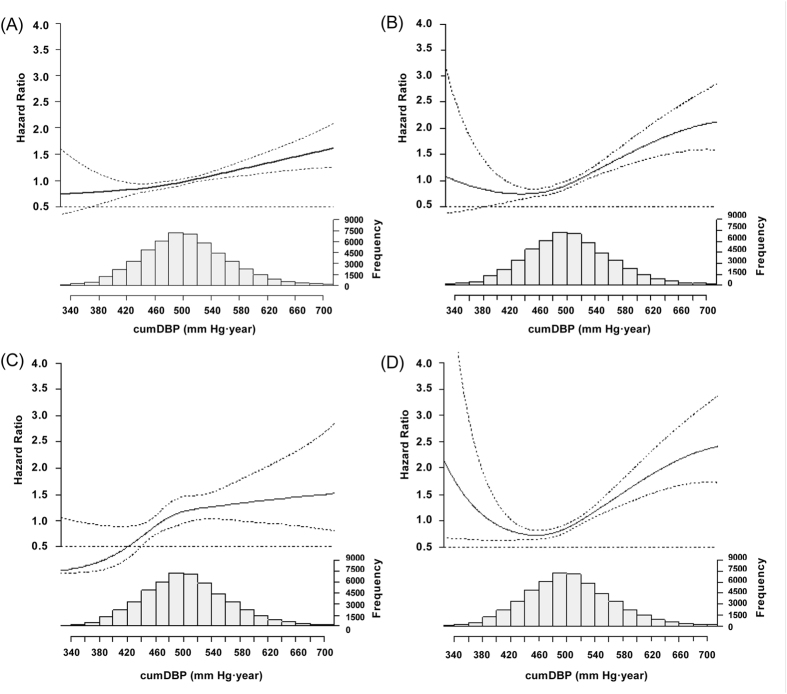
The Relationship Between cumDBP and Endpoint Events in the Study Population. (**A**) All-cause mortality, (**B**) Cardiovascular and cerebrovascular events, (**C**) Myocardial infarction, and (**D**) Stroke cumDBP, cumulative diastolic blood pressure; Freq, frequency.

Natural spline function analysis showed that for the study population, after adjusted for other confounders, cumSBP and cumDBP had a J-curve relationship with CCEs, and a U-curve relationship with stroke(ischemic stroke and hemorrhagic stroke) (Figs 2 and 3, supplementary Figs 2–5).

### Sensitivity Analysis

Using all-cause mortality, CCEs, stroke, or MI as the dependent variable, ScumSBP or ScumDBP as the independent variable, the risk for endpoint events was re-estimated. After adjusted for other confounders including baseline SBP and baseline DBP, similar results were found, indicating that ScumSBP (cumSBP) and ScumDBP (cumDBP) were risk factors for all-cause mortality, CCEs, and stroke (Supplementary Tables 1 and 2).

## Discussion

Hypertension is the most common chronic disease with stroke and MI as its most common complications that can lead to death^12^,^13^,^14^. BP is affected by various factors, such as age, lifestyle, and diet; therefore, a single measurement of BP cannot accurately predict CCEs and all-cause mortality. Cumulative exposure is determined by the level and duration of exposure and has been used to study smoking and lung cancer, hyperglycemia and complications of diabetes, hyperlipidemia and coronary disease, as well as BP and kidney damage^6^,^7^,^8^,^9^,^10^. In the current analysis, we found that for the study population, the cumulative incidence of all-cause mortality, CCEs, MI, and stroke increased with the increase in cumSBP (*P* < 0.05). Previously, it has been found that the population cumulative incidence of all-cause mortality increased with elevated SBP starting from 100 mm Hg^12^. Similarly, in a longitudinal study that included 1990 adults aged 30–62 years with a follow-up of over 38 years, the cumulative incidence of all-cause mortality and CCEs increased with the increase in SBP starting from 120 mm Hg^15^.

In the current analysis, we have found that after adjusted for confounding factors including baseline SBP and DBP, cumSBP and cumDBP remained risk factors for all-cause mortality, CCEs, and stroke; and cumSBP and cumDBP were better predictors for all-cause mortality, CCEs, and stroke than baseline SBP or baseline DBP. However, baseline SBP was found to be a risk factor for MI, while cumSBP, cumDBP, and baseline DBP had no predictive value for MI. Although baseline SBP had higher predictive value for MI than cumSBP and cumDBP, considering that the incidence of stroke among Chinese population is much higher than that of coronary heart disease, the overall predictive value of cumSBP and cumDBP for all-cause mortality and CCEs was better than that of baseline SBP and baseline DBP.

Studies have found that atherosclerosis is a complication due to long-term exposure to hypertension and other risk factors^16^. Constant high BP can increase the hemodynamic burden of the aorta, resulting in reduced arterial compliance and development of atherosclerosis. The changes in the arterial wall elasticity and function directly lead to various complications of atherosclerosis, including stroke, cardiovascular events or even death. These findings have provided a pathophysiological explanation for what we have found in the current analysis: increases in cumSBP and cumDBP resulted in increased risk for all-cause mortality, CCEs, and stroke.

Further analysis by natural spline function found a J-curve relationship between CCEs and cumulative BP (cumSBP and cumDBP) and a U-curve for stroke (ischemic stroke and hemorrhagic stroke) and cumulative BP (cumSBP and cumDBP). These findings indicated that with a too high or too low cumSBP or cumDBP, the risk for the occurrence of CCEs and stroke increased.

However, there were some limitations to this analysis, since the mean follow-up was < 4 years, which was relatively short, so that some of the endpoint events might not have sufficient time to develop. Although the impact of various confounding factors had been adjusted in our risk estimation models, other factors, such as duration of hypertension, white coat effect, drug compliance, the temperature and the environmental changes, remained unadjusted.

### Perspectives

In summary, we have found that with increases in cumSBP and cumDBP, the risk for all-cause mortality, cardiovascular and cerebrovascular events, and stroke also increased. Howard also found that even with successful treatment there is a substantial potential gain by prevention or delay of hypertension^17^. Compare with the standard treatment group (mean SBP:136.2 mm Hg). all-cause mortality was reduced 27% (95% CI, 10 to 40%; p = 0.003) by intensive BP treatment (mean SBP: 121.4 mm Hg)^18^. Therefore, for middle-aged and elder people who do not have hypertension, their blood pressure should be controlled within the desired range and should remain not elevated to keep cumSBP and cumDBP at a low level. For the management of hypertension in middle-aged and elder people, long-term effectiveness is as important as lowering the elevated blood pressure to within the normal range. The aim of blood pressure control is to reduce the risk for cardiovascular and cerebrovascular events and all-cause mortality caused by consistent high blood pressure, and to improve the quality of life of the patient.

cumSBP and cumDBP had better predictive value for endpoint events than baseline SBP and DBP using the same models, since cumulative exposure has evaluated the effect of exposure time as well as the exposure level, and is therefore a better predictor for the chronic effects of exposure on the target organs. Our study has also demonstrated that invaluable findings contributing to the understanding of human health can be made by gathering and analyzing data obtained from repetitive medical examinations. Following the usage of big data in more and more fields, it can be expected that collection of such cumulative exposure data will become simple and practical in the near future.

## Additional Information

**How to cite this article**: Wang, Y. X. *et al*. Predictive Value of Cumulative Blood Pressure for All-Cause Mortality and Cardiovascular Events. *Sci. Rep.* **7**, 41969; doi: 10.1038/srep41969 (2017).

**Publisher's note:** Springer Nature remains neutral with regard to jurisdictional claims in published maps and institutional affiliations.

## Supplementary Material

Supplementary Dataset 1

## Figures and Tables

**Table 1:** Characteristics of the Study Population by groups of cumSBP.

Variable	The first group (cumSBP <720) N = 16,361	The second group (720 ≤ cumSBP <780) N = 11,856	The third group (780 ≤ cumSBP <840) N = 9716	The fourth group (cumSBP ≥840) N = 14,452	Total (N = 52,385)	*P* value
Men, n (%)	10354 (63.3)	9774 (82.4)	8154 (83.9)	11859 (82.1)	40141 (76.6)	<0.001
Age, years	43.05 ± 10.54	47.12 ± 10.86	50.61 ± 10.84	55.56 ± 10.65	48.82 ± 11.77	<0.001
Heart rate, times/min	71.96 ± 9.20	73.14 ± 9.63	73.91 ± 9.97	74.70 ± 10.83	73.34 ± 9.96	<0.001
Baseline SBP, mm Hg	112.86 ± 11.71	123.62 ± 12.29	131.58 ± 14.03	146.52 ± 18.98	128.05 ± 19.64	<0.001
SBP (Visit 2), mm Hg	111.54 ± 9.95	123.93 ± 8.59	133.35 ± 9.36	151.45 ± 17.18	129.40 ± 19.72	<0.001
SBP (Visit 3), mm Hg	114.44 ± 10.97	126.18 ± 10.32	134.20 ± 11.70	149.65 ± 18.11	130.47 ± 19.13	<0.001
Baseline DBP, mm Hg	74.91 ± 8.22	80.93 ± 8.35	84.81 ± 9.31	90.63 ± 11.58	82.45 ± 11.30	<0.001
DBP (Visit 2), mm Hg	75.38 ± 7.50	82.34 ± 7.38	86.57 ± 8.30	93.22 ± 11.54	83.95 ± 11.31	<0.001
DBP (Visit 3), mm Hg	76.74 ± 7.95	83.13 ± 7.78	86.47 ± 8.70	91.93 ± 11.12	84.18 ± 10.80	<0.001
BMI, kg/m^2^	23.86 ± 3.25	25.04 ± 3.33	25.58 ± 3.41	26.05 ± 3.47	25.05 ± 3.47	<0.001
FBG, mmol/L	5.16 ± 1.17	5.35 ± 1.45	5.48 ± 1.60	5.61 ± 1.79	5.39 ± 1.52	<0.001
TC, mmol/L	4.78 ± 1.00	4.89 ± 1.15	4.98 ± 1.20	5.06 ± 1.20	4.92 ± 1.13	<0.001
HDL-C, mmol/L	1.51 ± 0.36	1.54 ± 0.39	1.57 ± 0.40	1.60 ± 0.42	1.55 ± 0.39	<0.001
LDL-C, mmol/L	2.25 ± 0.81	2.31 ± 0.86	2.32 ± 0.91	2.32 ± 1.02	2.30 ± 0.90	<0.001
eGFR(mL/min/1.73 m^2^)	88.06 ± 24.19	86.35 ± 26.58	83.37 ± 22.99	79.40 ± 25.90	84.41 ± 25.26	<0.001
salt intake, n (%)						
<6 gram/day, n (%)	1493 (9.4)	1049 (9.1)	879 (9.4)	1336 (9.7)	4757 (9.4)	0.001
6–10 gram/day, n (%)	12807 (80.7)	9266 (80.5)	7407 (79.3)	10944 (79.2)	40424 (80.0)	
≧ 10 gram/day, n (%)	1564 (9.9)	1193 (10.4)	1054 (11.3)	1539 (11.1)	5350 (10.6)	
Smoking, n (%)	4324 (26.4)	3854 (32.5)	3227 (33.2)	4307 (29.8)	15712 (30.0)	<0.001
Drinking, n (%)	1850 (11.3)	2064 (17.4)	2038 (21.0)	3228 (22.3)	9180 (17.5)	<0.001
Exercise, n (%)	1630 (10.0)	1324 (11.2)	1357 (14.0)	2763 (19.1)	7074 (13.5)	<0.001
Hypertension, n (%)	1189 (7.3)	3040 (25.6)	4599 (47.3)	11,108 (76.9)	19,936 (38.1)	<0.001
Antihypertensive drugs use, n (%)	212 (1.3)	433 (3.7)	806 (8.3)	2965 (20.5)	4416 (8.4)	<0.001
Lipid-lowering drugs use, n (%)	60 (0.4)	68 (0.6)	91 (0.9)	193 (1.3)	412 (0.8)	<0.001
Diabetes medications, n (%)	145 (0.9)	142 (1.2)	202 (2.1)	474 (3.3)	963 (1.8)	<0.001
atrial fibrillation, n (%)	35 (0.2)	40 (0.3)	41 (0.4)	100 (0.7)	216 (0.4)	<0.001

Values are mean ± SD or n (%). 1 mm Hg = 0.133 kPa.

BMI indicates body mass index; cumSBP, cumulative systolic blood pressure; DBP, diastolic blood pressure; eGFR, estimated glomerular filtration rate; FBG, fasting blood glucose; HDL-C, high-density lipoprotein cholesterol; LDL-C, low-density lipoprotein cholesterol; SBP, systolic blood pressure; and TC, total cholesterol.

**Table 2:** Endpoint Events by four groups of cumSBP.

	The first group (cumSBP < 720) N = 16,361	The second group (720≤ cumSBP < 780) N = 11,856	The third group (780 ≤ cumSBP < 840) N = 9716	The fourth group (cumSBP ≥ 840) N = 14,452	Total N = 52,385	Log-rank Test
All-cause mortality, n (%)	126 (0.8)	162 (1.4)	215 (2.2)	545 (3.8)	1048 (2.0)	<0.001
CV and cerebrovascular events, n (%)	69 (0.4)	80 (0.7)	138 (1.4)	373 (2.6)	660 (1.3)	<0.001
Myocardial infarction, n (%)	11 (0.1)	27 (0.2)	43 (0.4)	85 (0.6)	166 (0.3)	<0.001
Stroke, n (%)	58 (0.4)	53 (0.4)	95 (1.0)	291 (2.0)	497 (0.9)	<0.001

cumSBP indicates cumulative systolic blood pressure; CV, cardiovascular.

**Table 3:** cumSBP and Endpoint Events Calculated Using Cox Proportional Hazards Model.

Variable		All-cause mortality HR (95% CI)	CV and cerebrovascular events HR (95% CI)	Myocardial infarction HR (95% CI)	Stroke HR (95% CI)
Model 1	cumSBP (every 10 mm Hg·year increase)	1.019 (1.014, 1.024)*	1.035 (1.030, 1.041)*	1.026 (1.014, 1.038)*	1.038 (1.032, 1.045)*
Model 2	cumSBP (every 10 mm Hg·year increase)	1.015 (1.008, 1.022)*	1.018 (1.010, 1.027)*	1.011 (0.995, 1.029)	1.021 (1.012, 1.031)*
	Baseline SBP (every 1 mm Hg increase)	1.004 (1.000, 1.008)	1.011 (1.006, 1.016)*	1.011 (1.001, 1.021)†	1.010 (1.005, 1.016)*
Model 3	cumSBP (every 10 mm Hg·year increase)	1.013 (1.006, 1.021)*	1.018 (1.010, 1.027)*	1.013 (0.996, 1.030)	1.021 (1.011, 1.031)*
	Baseline SBP (every 1 mm Hg increase)	1.004 (1.000, 1.008)	1.011 (1.006, 1.016)*	1.012 (1.002, 1.022)†	1.011 (1.005, 1.017)*

Model 1: adjusted for sex and age.

Model 2: adjusted for model 1 and further adjusted for baseline SBP, BMI, FBG, HDL-C, exercise, smoking, drinking, and antihypertensive drugs use.

Model 3: adjusted for model 2 and further adjusted for salt intake, eGFR, lipid-lowering drugs use, diabetes medications, and number of antihypertensive medications.

BMI, body mass index; cumSBP, cumulative systolic blood pressure; CV, cardiovascular; eGFR, estimated glomerular filtration rate; FBG, fasting blood glucose; and HDL-C, high-density lipoprotein cholesterol.

^*^P < 0.01, ^†^*P* < 0.05.

**Table 4:** cumDBP and Endpoint Events Calculated Using Cox Proportional Hazards Model.

Variable		All-cause mortality HR (95% CI)	CV and cerebrovascular events HR (95% CI)	Myocardial infarction HR (95% CI)	Stroke HR (95% CI)
Model 1	cumDBP (every 5 mm Hg·year increase)	1.013 (1.008, 1.017)*	1.029 (1.024, 1.033)*	1.019 (1.009, 1.030)*	1.031 (1.026, 1.037)*
Model 2	cumDBP (every 5 mm Hg·year increase)	1.012 (1.006, 1.018)*	1.017 (1.010, 1.023)*	1.013 (1.000, 1.027)	1.018 (1.011, 1.026)*
	Baseline DBP (every 1 mm Hg increase)	1.003 (0.996, 1.010)	1.017 (1.009, 1.025)*	1.006 (0.989, 1.023)	1.020 (1.010, 1.029)*
Model 3	cumDBP (every 5 mm Hg·year increase)	1.012 (1.006, 1.018)*	1.017 (1.010, 1.024)*	1.015 (1.000, 1.029)	1.018 (1.010, 1.026)*
	Baseline DBP (every 1 mm Hg increase)	1.002 (0.995, 1.010)	1.017 (1.009, 1.026)*	1.007 (0.990, 1.024)	1.020 (1.010, 1.029)*

Model 1: adjusted for sex and age.

Model 2: adjusted for model 1 and further adjusted for baseline DBP, BMI, FBG, HDL-C, exercise, smoking, drinking, and antihypertensive drugs use.

Model 3: adjusted for model 2 and further adjusted for salt intake, eGFR, lipid-lowering drugs use, diabetes medications, and number of antihypertensive medications.

BMI, body mass index; cumDBP, cumulative diastolic blood pressure; CV, cardiovascular; eGFR, estimated glomerular filtration rate; FBG, fasting blood glucose; and HDL-C, high-density lipoprotein cholesterol.

^*^P < 0.01.

**Table 5:** Predictive Value of the Cox Regression Models.

	All-cause mortality		CV and cerebrovascular events		Myocardial infarction		Stroke	
	LR *X*^2^	*P* value	LR *X*^2^	*P* value	LR *X*^2^	*P* value	LR *X*^2^	*P* value
cumSBP added to model 3	1089.66	<0.001	498.10	>0.05	127.72	>0.05	419.22	<0.01
Baseline SBP added to model 3	1077.05		498.43		131.08		411.76	
cumDBP added to model 3	1061.55	>0.05	469.43	<0.01	120.09	>0.05	398.14	<0.05
Baseline DBP added to model 3	1058.07		460.30		116.73		391.39	

Model 3: adjusted for sex, age, BMI, FBG, HDL-C, salt intake, exercise, smoking, drinking, antihypertensive drugs use, eGFR, lipid-lowering drugs use, diabetes medications, and number of antihypertensive medications.

BMI, body mass index; cumDBP, cumulative diastolic blood pressure; CV, cardiovascular; eGFR, estimated glomerular filtration rate; FBG, fasting blood glucose; HDL-C, high-density lipoprotein cholesterol; and LR, likelihood ratio.

## References

[b1] Banach M. . Association of systolic blood pressure levels with cardiovascular events and all-cause mortality among older adults taking antihypertensive medication. Int J Cardiol. 176, 219–226 (2014). 25085381 10.1016/j.ijcard.2014.07.067 PMC4144437

[b2] Triantafyllou A. . Accumulation of microvascular target organ damage in newly diagnosed hypertensive patients. Journal of the American Society of Hypertension. 8, 542–549 (2014). 24913569 10.1016/j.jash.2014.04.008

[b3] Lewington S. . Age-specific relevance of usual blood pressure to vascular mortality: A meta-analysis of individual data for one million adults in 61 prospective studies. Lancet. 360, 1903–1913 (2002). 12493255 10.1016/s0140-6736(02)11911-8

[b4] Dorresteijn J. A. . Relation between blood pressure and vascular events and mortality in patients with manifest vascular disease: J-curve revisited. Hypertension. 59, 14–21 (2011). 22068865 10.1161/HYPERTENSIONAHA.111.179143

[b5] NIH. Syntax of referencing in The Framingham Study, (ed. BUSHEW) 74–599 (Bethesda: N.I.H., 1968).

[b6] Doll R. & Hill A. B. Smoking and carcinoma of the lung. BMJ. 2, 739–748 (1950). 14772469 10.1136/bmj.2.4682.739 PMC2038856

[b7] Doll R. & Hill A. B. Study of the aetiology of carcinoma of the lung. BMJ. 2, 1271–1286 (1952). 12997741 10.1136/bmj.2.4797.1271 PMC2022425

[b8] Prospective U. K. diabetes study 16. Overview of 6 years’ therapy of type ii diabetes: A progressive disease. U.K. Prospective diabetes study group. Diabetes. 44, 1249–1258 (1995). 7589820

[b9] Navar-Boggan A. M. . Hyperlipidemia in early adulthood increases long-term risk of coronary heart disease. Circulation. 131, 451–458 (2015). 25623155 10.1161/CIRCULATIONAHA.114.012477 PMC4370230

[b10] Zemaitis P. . Cumulative systolic bp and changes in urine albumin-to-creatinine ratios in nondiabetic participants of the multi-ethnic study of atherosclerosis. Clinical Journal of the American Society of Nephrology. 9, 1922–1929 (2014). 25200476 10.2215/CJN.02450314 PMC4220754

[b11] Wu S. . Prevalence of ideal cardiovascular health and its relationship with the 4-year cardiovascular events in a northern chinese industrial city. Circulation: Cardiovascular Quality and Outcomes. 5, 487–493 (2012). 22787064 10.1161/CIRCOUTCOMES.111.963694

[b12] Port S. . Systolic blood pressure and mortality. The Lancet. 355, 175–180 (2000). 10.1016/S0140-6736(99)07051-8 10675116

[b13] JATOS Study Group. Principal results of the japanese trial to assess optimal systolic blood pressure in elderly hypertensive patients (jatos). Hypertension Research. 31, 2115–2127 (2008). 19139601 10.1291/hypres.31.2115

[b14] Ogihara T. . Target blood pressure for treatment of isolated systolic hypertension in the elderly: Valsartan in elderly isolated systolic hypertension study. Hypertension. 56, 196–202 (2010). 20530299 10.1161/HYPERTENSIONAHA.109.146035

[b15] Kannel W. B., D’Agostino R. B. & Silbershatz H. Blood pressure and cardiovascular morbidity and mortality rates in the elderly. American Heart Journal. 134, 758–763 (1997). 9351745 10.1016/s0002-8703(97)70061-9

[b16] Mitchell G. F. Arterial stiffness and hypertension. Hypertension. 64, 13–18 (2014). 24752432 10.1161/HYPERTENSIONAHA.114.00921 PMC4063409

[b17] Howard G. . Is blood pressure control for stroke prevention the correct goal? The lost opportunity of preventing hypertension. Stroke. 46, 1595–1600 (2015). 25953369 10.1161/STROKEAHA.115.009128 PMC4924927

[b18] Banach M. . blood pressure and kidney update 2015. Lipids Health Dis. 14, 167 (2015). 26718096 10.1186/s12944-015-0169-0 PMC4696333

