# Supplementary material for: Predictive Value of Cumulative Blood Pressure for All-Cause Mortality and Cardiovascular Events
Source: Sci Rep. 2017 Feb 7;7:41969. doi: 10.1038/srep41969 (PMC5294637; doi:10.1038/srep41969)
Supplement: Supplementary Dataset 1 [file srep41969-s1.docx]

**Predictive Value of Cumulative Blood Pressure for All-Cause Mortality and Cardiovascular Events**

Yan Xiu Wang^1,2^*, Lu Song^2,3^*, Ai Jun Xing^2^, Ming Gao^2^, Hai Yan Zhao^2^,Chun Hui Li ^2,3^,Hua Ling Zhao^2,3^, Shuo Hua Chen^4^, Cheng Zhi Lu^1^*, Shou Ling Wu^2^*

^1^Department of Cardiology,Tianjin First Center Hospital, Clinical Medical College of Tianjin Medical University, Tianjin, China

^2^ Department of Cardiology, Kailuan Hospital, North China University of Science and Technology, Tangshan, China

^3^ Graduate school, North China University of Science and Technology, Tangshan, China

^4^Department of Health Care Center, Kailuan Hospital, North China University of Science and Technology, Tangshan, China

*Yan Xiu Wang and Lu Song contributed to this work and share the first authorship. Shou Ling Wu and Cheng Zhi Lu share the last authorship.

Corresponding authors: Shouling Wu, Department of Cardiology, Kailuan Hospital , North China University of Science and Technology, China, Tangshan, 063000, Tel: +86-315-3025655; fax: +86-315-3025655; e-mail: [drwusl@163.com](mailto:drwusl@163.com); Cheng Zhi Lu, Department of Cardiology,Tianjin First Center Hospital, Clinical Medical College of Tianjin Medical University, China, Tianjin, 300000,e-mail：[lucz8@126.com](mailto:biochemgao@163.com)

101,510 adults participated the Kailuan study in 2006

57,927 participants accept the second and the third medical examinations in 2008–2009, and 2010–2011.

Excluding 1,751 participants without BP data in 2006, 2008 or 2010

Excluding 3,791 participants with MI, or Stroke in or prior to 2011

52,385 participants for analysis

Supplement figure 1: Flowchart of the participants included in the current analysis

**
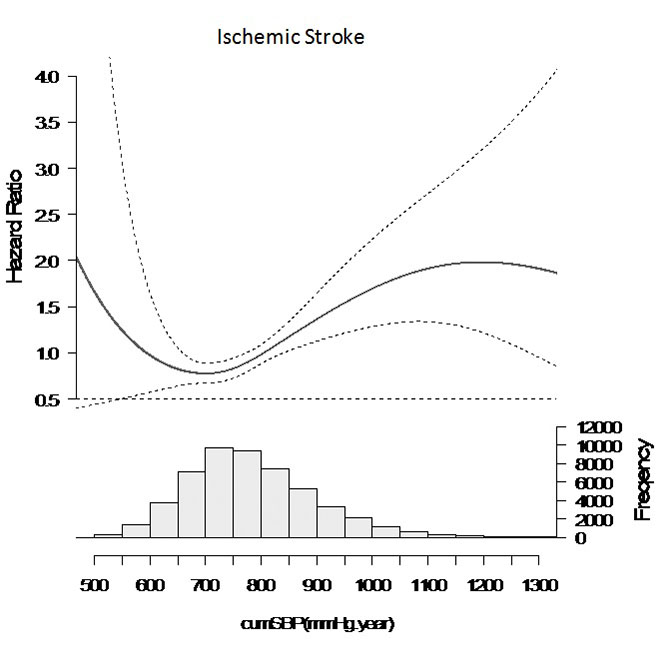
**

**Supplement Figure 2.** The Relationship between cumSBP and Ischemic stroke Stroke in the Study Population

cumSBP, cumulative systolic blood pressure. Freq, frequency.


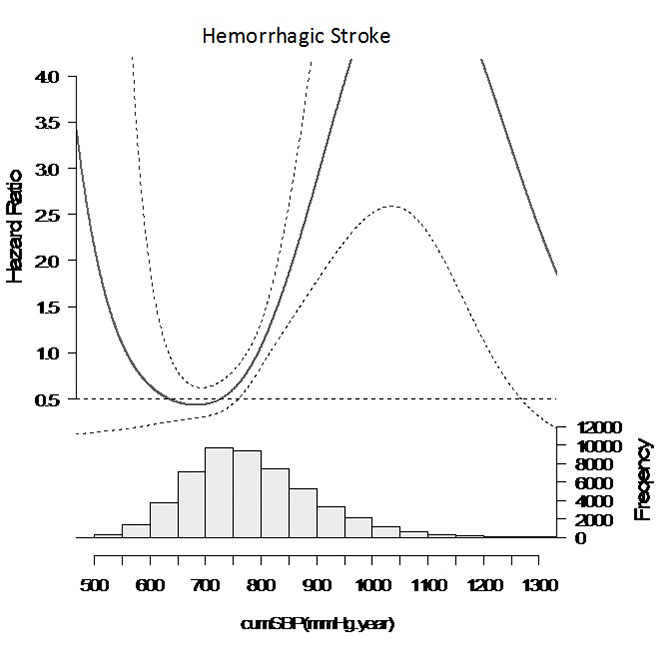


**Supplement Figure 3.** The Relationship between cumSBP and Hemorrhagic Stroke in the Study Population

cumSBP,cumulative systolic blood pressure. Freq, frequency.


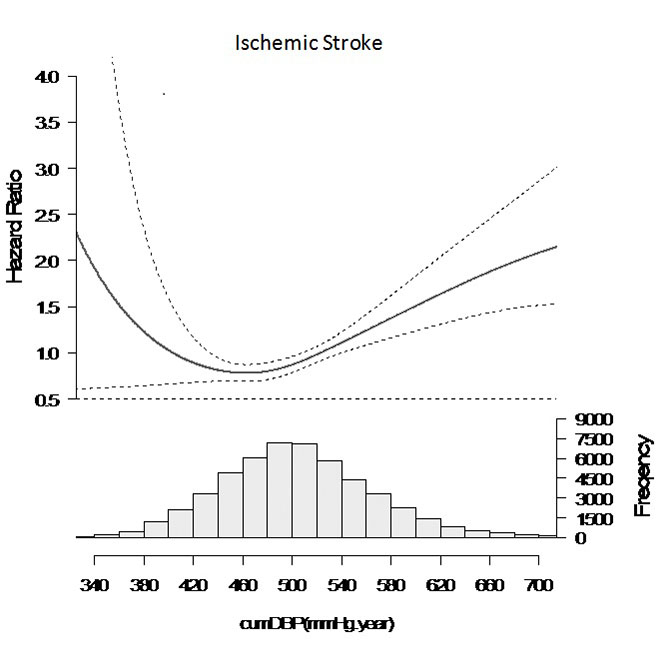


**Supplement Figure 4.** The Relationship between cumDBP and Ischemic stroke Stroke in the Study Population

cumDBP, cumulative diastolic blood pressure. Freq, frequency.


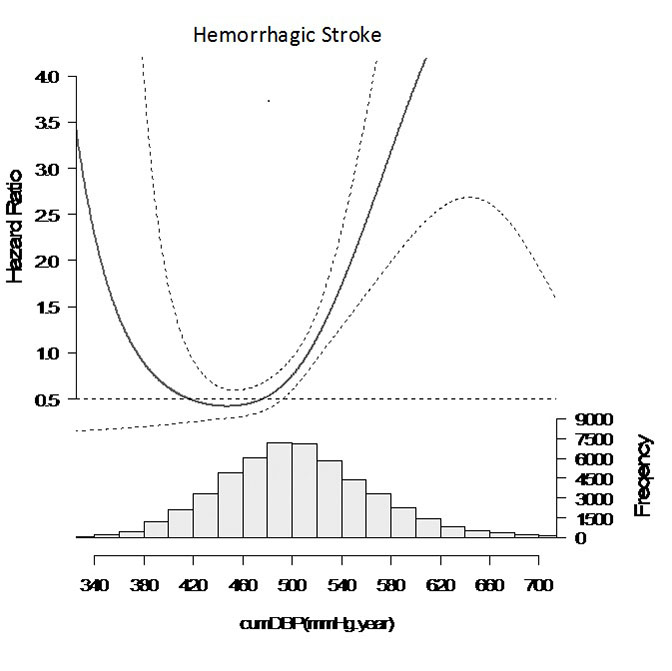


**Supplement Figure 5.** The Relationship between cumDBP and Hemorrhagic Stroke in the Study Population

cumDBP, cumulative diastolic blood pressure; Freq, frequency.

**Supplementary Table 1. ScumSBP and Endpoint Events Calculated Using Cox Proportional Hazards Model, HR (95% CI)**

| Variable | | All-cause mortality  HR (95%CI) | Cardiovascular and cerebrovascular events  HR (95%CI) | Myocardial infarction  HR (95%CI) | Stroke  HR (95%CI) |
| --- | --- | --- | --- | --- | --- |
| Model 1 | ScumSBP (every 10 mm Hg·year increase) | 1.129 (1.090, 1.169)* | 1.325 (1.271, 1.380)* | 1.206 (1.106, 1.314)* | 1.362 (1.300, 1.426)* |
| Model 2 | ScumSBP (every 10 mm Hg·year increase) | 1.095 (1.037, 1.156)^†^ | 1.204 (1.129, 1.285)* | 1.071 (0.936, 1.226) | 1.247 (1.159, 1.342)* |
|  | Baseline SBP (every 1 mm Hg increase) | 1.004 (0.999, 1.008) | 1.007 (1.001, 1.012)^†^ | 1.011 (1.001, 1.022)^†^ | 1.005 (0.999, 1.011) |
| Model 3 | ScumSBP (every 10 mm Hg·year increase) | 1.087 (1.026, 1.151)^†^ | 1.204 (1.126, 1.287)* | 1.082 (0.942, 1.242) | 1.243 (1.153, 1.341)* |
|  | Baseline SBP (every 1 mm Hg increase) | 1.004 (0.999, 1.008) | 1.007 (1.002, 1.013)^†^ | 1.012 (1.001, 1.024)^†^ | 1.006 (0.999, 1.012) |

Model 1: adjusted for sex and age.

Model 2: adjusted for model 1 and further adjusted for baseline SBP, BMI, FBG, HDL-C, exercise, smoking, drinking, and antihypertensive drugs use.

Model 3: adjusted for model 2 and further adjusted for salt intake, eGFR, lipid-lowering drugs use, diabetes medications, and number of antihypertensive medications.

BMI, body mass index; cumSBP, cumulative systolic blood pressure; CV, cardiovascular; eGFR, estimated glomerular filtration rate; FBG, fasting blood glucose; and HDL-C, high-density lipoprotein cholesterol.

**P*<0.01, ^†^*P*<0.05.

**Supplementary Table 2. ScumDBP and Endpoint Events Calculated Using Cox Proportional Hazards Model**

| Variable | | All-cause mortality  HR (95%CI) | Cardiovascular and cerebrovascular events  HR (95%CI) | Myocardial infarction  HR (95%CI) | Stroke  HR (95%CI) |
| --- | --- | --- | --- | --- | --- |
| Model 1 | ScumDBP (every 5 mm Hg·year increase) | 1.124 (1.090, 1.159)* | 1.312 (1.267, 1.358)* | 1.191 (1.106, 1.282)* | 1.346 (1.295, 1.400)* |
| Model 2 | ScumDBP (every 5 mm Hg·year increase) | 1.082 (1.047, 1.119)* | 1.287 (1.240, 1.335)* | 1.019 (1.009, 1.030)* | 1.326 (1.272, 1.382)* |
| Model 3 | ScumDBP (every 5 mm Hg·year increase) | 1.084 (1.031, 1.140)^†^ | 1.203 (1.137, 1.274)* | 1.125 (1.000, 1.265) | 1.227 (1.150, 1.310)* |
|  | Baseline DBP (every 1 mm Hg increase) | 1.001 (0.993, 1.010) | 1.009 (0.999, 1.018) | 1.005 (0.986, 1.024) | 1.010 (0.999, 1.021) |

Model 1: adjusted for sex and age.

Model 2: adjusted for model 1 and further adjusted for baseline DBP, BMI, FBG, HDL-C, exercise, smoking, drinking, and antihypertensive drugs use.

Model 3: adjusted for model 2 and further adjusted for salt intake, eGFR, lipid-lowering drugs use, diabetes medications, and number of antihypertensive medications.

BMI, body mass index; cumDBP, cumulative diastolic blood pressure; CV, cardiovascular; eGFR, estimated glomerular filtration rate; FBG, fasting blood glucose; and HDL-C, high-density lipoprotein cholesterol.

**P*<0.01 ^†^*P*<0.05.

**Supplementary Table 3. cumSBP and Endpoint Events Calculated Using Cox Proportional Hazards Model (nonhypertension=32,449)**

| Variable | | All-cause mortality  HR (95%CI) | CV and cerebrovascular events  HR (95%CI) | Myocardial infarction  HR (95%CI) | Stroke  HR (95%CI) |
| --- | --- | --- | --- | --- | --- |
| Model 1 | cumSBP (every 10 mm Hg·year increase) | 1.022 (1.011, 1.032)* | 1.034 (1.020, 1.048)* | 1.040 (1.013, 1.067)† | 1.033 (1.017, 1.050)* |
| Model 2 | cumSBP (every 10 mm Hg·year increase) | 1.021 (1.009, 1.034)* | 1.028 (1.012, 1.044)† | 1.035 (1.004, 1.066)† | 1.028 (1.018, 1.047)† |
|  | Baseline SBP (every 1 mm Hg increase) | 1.001 (0.991, 1.011) | 1.004 (0.990, 1.018) | 1.015 (0.986, 1.044) | 0.999 (0.983, 1.016) |
| Model 3 | cumSBP (every 10 mm Hg·year increase) | 1.021 (1.008, 1.033)* | 1.030 (1.013, 1.046)† | 1.040 (1.010, 1.071)† | 1.029 (1.009, 1.048)† |
|  | Baseline SBP (every 1 mm Hg increase) | 1.000 (0.989, 1.011) | 1.005 (0.990, 1.020) | 1.014 (0.985, 1.045) | 1.000 (0.984, 1.017) |

Model 1: adjusted for sex and age.

Model 2: adjusted for model 1 and further adjusted for baseline SBP, BMI, FBG, HDL-C, exercise, smoking, drinking, and antihypertensive drugs use.

Model 3: adjusted for model 2 and further adjusted for salt intake, eGFR, lipid-lowering drugs use, diabetes medications, and number of antihypertensive medications.

BMI, body mass index; cumSBP, cumulative systolic blood pressure; CV, cardiovascular; eGFR, estimated glomerular filtration rate; FBG, fasting blood glucose; HDL-C, high-density lipoprotein cholesterol; and SBP, systolic blood pressure.

**P*<0.01, ^†^*P*<0.05.

**Supplementary Table 4. cumDBP and Endpoint Events Calculated Using Cox Proportional Hazards Model (nonhypertension = 32,449)**

| Variable | | All-cause mortality  HR (95%CI) | CV and cerebrovascular events  HR (95%CI) | Myocardial infarction  HR (95%CI) | Stroke  HR (95%CI) |
| --- | --- | --- | --- | --- | --- |
| Model 1 | cumDBP (every 5 mm Hg·year increase) | 1.012 (1.003, 1.021)^†^ | 1.031 (1.020, 1.042)* | 1.036 (1.015, 1.058)^†^ | 1.030 (1.017, 1.044)* |
| Model 2 | cumDBP (every 5 mm Hg·year increase) | 1.017 (1.007, 1.027)^†^ | 1.026 (1.013, 1.039)* | 1.034 (1.010, 1.057)^†^ | 1.024 (1.009, 1.040)* |
|  | Baseline DBP (every 1 mm Hg increase) | 0.990 (0.974, 1.005) | 1.009 (0.986, 1.033) | 1.022 (0.976, 1.071) | 1.005 (0.978, 1.031) |
| Model 3 | cumDBP (every 5 mm Hg·year increase) | 1.017 (1.006, 1.027)^†^ | 1.027 (1.014, 1.040)* | 1.038 (1.015, 1.061)^†^ | 1.025 (1.009, 1.040)* |
|  | Baseline DBP (every 1 mm Hg increase) | 0.991 (0.975, 1.007) | 1.010 (0.986, 1.034) | 1.022 (0.974, 1.073) | 1.006 (0.979, 1.033) |

Model 1: adjusted for sex and age.

Model 2: adjusted for model 1 and further adjusted for baseline DBP, BMI, FBG, HDL-C, exercise, smoking, drinking, and antihypertensive drugs use.

Model 3: adjusted for model 2 and further adjusted for salt intake, eGFR, lipid-lowering drugs use, diabetes medications, and number of antihypertensive medications.

BMI, body mass index; cumDBP, cumulative diastolic blood pressure; CV, cardiovascular; eGFR, estimated glomerular filtration rate; FBG, fasting blood glucose; and HDL-C, high-density lipoprotein cholesterol.

**P*<0.01, ^†^*P*<0.05.

**Supplementary Table 5. cumSBP and Endpoint Events Calculated Using Cox Proportional Hazards Model (Excluding Those Who Died Within 1 year of the Third Medical Examination, N=52,162)**

| Variable | | All-cause mortality  HR (95%CI) | CV and cerebrovascular events  HR (95%CI) | Myocardial infarction  HR (95%CI) | Stroke  HR (95%CI) |
| --- | --- | --- | --- | --- | --- |
| Model 1 | cumSBP (every 10 mm Hg·year increase) | 1.019 (1.014, 1.025)* | 1.035 (1.029, 1.041)* | 1.025 (1.012, 1.037)* | 1.038 (1.032, 1.045)* |
| Model 2 | cumSBP (every 10 mm Hg·year increase) | 1.016 (1.009, 1.024)* | 1.018 (1.009, 1.026)* | 1.010 (0.993, 1.027) | 1.021 (1.012, 1.030)* |
|  | Baseline SBP (every 1 mm Hg increase) | 1.003 (0.998, 1.007) | 1.011 (1.006, 1.016)* | 1.011 (1.001, 1.021)^†^ | 1.011 (1.005, 1.017)* |
| Model 3 | cumSBP (every 10 mm Hg·year increase) | 1.015 (1.007, 1.023)* | 1.018 (1.010, 1.027)* | 1.013 (0.995, 1.030) | 1.021 (1.011, 1.030)* |
|  | Baseline SBP (every 1 mm Hg increase) | 1.003 (0.998, 1.008) | 1.012 (1.007, 1.017)* | 1.011 (1.001, 1.022)^†^ | 1.011 (1.006, 1.017)* |

Model 1: adjusted for sex and age.

Model 2: adjusted for model 1 and further adjusted for baseline SBP, BMI, FBG, HDL-C, exercise, smoking, drinking, and antihypertensive drugs use.

Model 3: adjusted for model 2 and further adjusted for salt intake, eGFR, lipid-lowering drugs use, diabetes medications, and number of antihypertensive medications.

BMI, body mass index; cumSBP, cumulative systolic blood pressure; CV, cardiovascular; eGFR, estimated glomerular filtration rate; FBG, fasting blood glucose; and HDL-C, high-density lipoprotein cholesterol.

**P*<0.01, ^†^*P*<0.05.

**Supplementary Table 6. cumDBP and Endpoint Events Calculated Using Cox Proportional Hazards Model (Excluding Those Who Died Within 1 year of the Third Medical Examination, N = 52,162)**

| Variable | | All-cause mortality  HR (95%CI) | CV and cerebrovascular events  HR (95%CI) | Myocardial infarction  HR (95%CI) | Stroke  HR (95%CI) |
| --- | --- | --- | --- | --- | --- |
| Model 1 | cumDBP (every 5 mm Hg·year increase) | 1.013 (1.008, 1.018)* | 1.029 (1.024, 1.034)* | 1.019 (1.008, 1.029)^†^ | 1.032 (1.026, 1.037)* |
| Model 2 | cumDBP (every 5 mm Hg·year increase) | 1.013 (1.006, 1.020)* | 1.017 (1.010, 1.023)* | 1.013 (0.999, 1.027) | 1.018 (1.011, 1.026)* |
|  | Baseline DBP (every 1 mm Hg increase) | 1.001 (0.993, 1.009) | 1.018 (1.010, 1.026)* | 1.007 (0.990, 1.024) | 1.021 (1.012, 1.030)* |
| Model 3 | cumDBP (every 5 mm Hg·year increase) | 1.014 (1.007, 1.021)* | 1.017 (1.010, 1.024)* | 1.015 (1.000, 1.029) | 1.018 (1.010, 1.026)* |
|  | Baseline DBP (every 1 mm Hg increase) | 1.000 (0.991, 1.008) | 1.018 (1.010, 1.027)* | 1.007 (0.990, 1.025) | 1.021 (1.012, 1.031)* |

Model 1: adjusted for sex and age.

Model 2: adjusted for model 1 and further adjusted for baseline DBP, BMI, FBG, HDL-C, exercise, smoking, drinking, and antihypertensive drugs use.

Model 3: adjusted for model 2 and further adjusted for slat intake, eGFR, lipid-lowering drugs use, diabetes medications, and number of antihypertensive medications.

BMI, body mass index; cumDBP, cumulative diastolic blood pressure; CV, cardiovascular; eGFR, estimated glomerular filtration rate; FBG, fasting blood glucose; and HDL-C, high-density lipoprotein cholesterol.

**P*<0.01, ^†^*P*<0.05.

**Supplementary Table 7. cumSBP/cumDBP and Endpoint Events Calculated Using Cox Proportional Hazards Model in the participants without atrial fibrillation (n=52,169)**

| Variable | | All-cause mortality  HR (95%CI) | CV and cerebrovascular events  HR (95%CI) | Myocardial infarction  HR (95%CI) | Stroke  HR (95%CI) |
| --- | --- | --- | --- | --- | --- |
| Model 3 | cumSBP (every 10 mm Hg·year increase) | 1.013 (1.005, 1.020)* | 1.018 (1.010, 1.027)* | 1.012 (0.995, 1.030) | 1.021 (1.011, 1.031)* |
|  | Baseline SBP (every 1 mm Hg increase) | 1.004 (1.000, 1.008) | 1.012 (1.007, 1.017)* | 1.012 (1.002, 1.022)^†^ | 1.011 (1.005, 1.017)* |
| Model 3* | cumDBP (every 5 mm Hg·year increase) | 1.011(1.005, 1.018)* | 1.017 (1.010, 1.023)* | 1.015 (1.001, 1.028)^†^ | 1.018(1.010, 1.026)* |
|  | Baseline DBP (every 1 mm Hg increase) | 1.003 (0.995, 1.010) | 1.017 (1.009, 1.026)* | 1.007 (0.990, 1.025) | 1.020 (1.010, 1.030)* |

Model 3: adjusted for sex and age. eGFR, baseline SBP, BMI, FBG, HDL-C,salt intake, exercise, smoking, drinking, and antihypertensive drugs use, lipid-lowering drugs use, diabetes medications, and number of antihypertensive medications.

Model 3*: adjusted for sex and age. eGFR, baseline DBP, BMI, FBG, HDL-C, salt intake, exercise, smoking, drinking, and antihypertensive drugs use, lipid-lowering drugs use, diabetes medications, and number of antihypertensive medications.

BMI, body mass index; cumSBP, cumulative systolic blood pressure; CV, cardiovascular; eGFR, estimated glomerular filtration rate; FBG, fasting blood glucose; and HDL-C, high-density lipoprotein cholesterol.

**P*<0.01, ^†^*P*<0.05.

**Supplementary Table 8. cumSBP/cumDBP and Endpoint Events Calculated Using Cox Proportional Hazards Model in untreated hypertensive (n=15,520)**

| Variable | | All-cause mortality  HR (95%CI) | CV and cerebrovascular events  HR (95%CI) | Myocardial infarction  HR (95%CI) | Stroke  HR (95%CI) |
| --- | --- | --- | --- | --- | --- |
| Model 3 | cumSBP (every 10 mm Hg·year increase) | 1.008 (0.997, 1.019) | 1.013 (1.001, 1.025)**^†^** | 1.002 (0.977, 1.027) | 1.017 (1.003, 1.030)**^†^** |
|  | Baseline SBP (every 1 mm Hg increase) | 1.007 (1.000, 1.015) | 1.016 (1.008, 1.024)* | 1.010 (0.994, 1.027) | 1.017 (1.008, 1.026)* |
| Model 3* | cumDBP (every 5 mm Hg·year increase) | 1.008(0.998, 1.017) | 1.012 (1.003, 1.022)**^†^** | 1.003 (0.983, 1.024) | 1.015(1.004, 1.025)**^†^** |
|  | Baseline DBP (every 1 mm Hg increase) | 0.997 (0.984, 1.010) | 1.020 (1.006, 1.034)**^†^** | 0.991 (0.962, 1.020) | 1.028 (1.012, 1.044)**^†^** |

Model 3: adjusted for sex and age, eGFR, baseline SBP, BMI, FBG, HDL-C, salt intake, exercise, smoking, drinking, lipid-lowering drugs use, diabetes medications.

Model 3*: adjusted for sex and age, eGFR, baseline DBP, BMI, FBG, HDL-C, salt intake, exercise, smoking, drinking, and antihypertensive drugs use, lipid-lowering drugs use, diabetes medications, and number of antihypertensive medications.

BMI, body mass index; cumSBP, cumulative systolic blood pressure; CV, cardiovascular; eGFR, estimated glomerular filtration rate; FBG, fasting blood glucose; and HDL-C, high-density lipoprotein cholesterol.

**P*<0.01, ^†^*P*<0.05.
